# Dynamic and Volumetric Variables Reliably Predict Fluid Responsiveness in a Porcine Model with Pleural Effusion

**DOI:** 10.1371/journal.pone.0056267

**Published:** 2013-02-13

**Authors:** Ole Broch, Matthias Gruenewald, Jochen Renner, Patrick Meybohm, Jan Schöttler, Katharina Heß, Markus Steinfath, Berthold Bein

**Affiliations:** 1 Department of Anesthesiology and Intensive Care Medicine, University Hospital Schleswig-Holstein, Campus Kiel, Schleswig-Holstein, Germany; 2 Department of Anesthesiology, Intensive Care Medicine and Pain Therapy, University Hospital Frankfurt, Hessen, Germany; 3 Department of Cardiothoracic and Vascular Surgery, University Hospital Schleswig-Holstein, Campus Kiel, Schleswig-Holstein, Germany; 4 Christian-Albrechts-University Kiel, Schleswig-Holstein, Germany; University of Leicester, United Kingdom

## Abstract

**Background:**

The ability of stroke volume variation (SVV), pulse pressure variation (PPV) and global end-diastolic volume (GEDV) for prediction of fluid responsiveness in presence of pleural effusion is unknown. The aim of the present study was to challenge the ability of SVV, PPV and GEDV to predict fluid responsiveness in a porcine model with pleural effusions.

**Methods:**

Pigs were studied at baseline and after fluid loading with 8 ml kg^−1^ 6% hydroxyethyl starch. After withdrawal of 8 ml kg^−1^ blood and induction of pleural effusion up to 50 ml kg^−1^ on either side, measurements at baseline and after fluid loading were repeated. Cardiac output, stroke volume, central venous pressure (CVP) and pulmonary occlusion pressure (PAOP) were obtained by pulmonary thermodilution, whereas GEDV was determined by transpulmonary thermodilution. SVV and PPV were monitored continuously by pulse contour analysis.

**Results:**

Pleural effusion was associated with significant changes in lung compliance, peak airway pressure and stroke volume in both responders and non-responders. At baseline, SVV, PPV and GEDV reliably predicted fluid responsiveness (area under the curve 0.85 (p<0.001), 0.88 (p<0.001), 0.77 (p = 0.007). After induction of pleural effusion the ability of SVV, PPV and GEDV to predict fluid responsiveness was well preserved and also PAOP was predictive. Threshold values for SVV and PPV increased in presence of pleural effusion.

**Conclusions:**

In this porcine model, bilateral pleural effusion did not affect the ability of SVV, PPV and GEDV to predict fluid responsiveness.

## Introduction

Several studies demonstrated that an individually tailored fluid therapy during major surgery was associated with reduced morbidity and length of stay on the intensive care unit by avoiding both fluid overloading and inappropriate application of vasoactive agents [1]. Furthermore, recent investigations reported long-term beneficial effects following goal-directed therapy [2]. However, numerous studies demonstrated static variables of preload, such as central venous pressure (CVP) and pulmonary occlusion pressure (PAOP) to be poor predictors of fluid responsiveness [3]. In some studies, static volumetric parameters such as global end-diastolic volume (GEDV) have been shown to reflect preload, their ability to indicate fluid responsiveness, however, remains controversial [4]–[6]. In contrast, the reliability of dynamic variables such as stroke volume variation (SVV) and pulse pressure variation (PPV) to indicate fluid responsiveness has been demonstrated repeatedly in various patient populations [7]–[10]. Several confounders like arrhythmia or vasoactive agents have been identified to impede the ability of these dynamic variables to predict fluid responsiveness [10]. The relationship between aortic impedance and intrathoracic pressure was investigated by several studies dealing with ventilation induced dynamic variables during open-chest conditions [11], [12]. The authors demonstrated an inverse relationship between aortic impedance and intrathoracic pressure and emphasized the alteration of stroke volume and its surrogate variable PPV in presence of open-chest, closed pericardium conditions.

Recently, SVV and PPV have been incorporated into advanced algorithms for performing goal-directed therapy [13], [14]. However, as these variables are based on the ventilation induced variations of arterial pressure and stroke volume, the reliability of SVV and PPV may be confounded by pleural effusions. In this context, recent investigations reported an incidence of pleural effusions in up to 60% of ICU patients, often diagnosed purely by chance [15], [16]. To date there are no data investigating the reliability of dynamic and volumetric variables of fluid responsiveness in presence of pleural effusion.

The aim of our prospective animal study was to determine the ability of SVV, PPV and GEDV to predict a percentage change ≥15% in stroke volume by pulmonary thermodilution (ΔSV_PAC_) in a porcine model with pleural effusion. We hypothesized that SVV, PPV and GEDV are still able to reliably predict fluid responsiveness under these circumstances, but that threshold values are affected by pleural effusion.

## Materials and Methods

After approval by the Animal Investigation Committee, Christian-Albrechts University Kiel (Permit Number: V 312-72241.121-39), the study was conducted in compliance with the recommendations in the Guide for the Care and Use of Laboratory Animals of the National Institutes of Health. The study was carried out in consideration of the Utstein-style guidelines on healthy swine (German domestic pigs), ranging from 12 to 16 weeks of age, weighing 34±2 kg and of either gender. The animals originate from the Institution for animal breeding, Christian-Albrechts University Kiel, Olshausen- straße 40, 24098 Kiel.

All surgery was performed under general anesthesia with the aim of avoiding pain and minimizing distress or suffering for the animals. The animals were fasted overnight, but had free access to water. At test day, premedication was performed with the neuropleptic azaperone (4 mg kg^−1^) 1 hour before surgery, and each animal was transferred directly to the operating theatre with the requirements of animal welfare during transport. Anesthesia was induced with a bolus dose of intramuscular ketamine (20 mg kg^−1^). After establishing venous access, propofol (2 mg kg^−1^) and sufentanil (0.5 µg kg^−1^) were administered via an ear vein. Airway management implied endotracheal intubation and pigs were ventilated with the Viasys Avea ventilator (Viasys Healthcare, Conshohocken, PA) in a volume-controlled mode with a tidal volume of 10 ml kg^−1^, a positive end-expiratory pressure of 5 cm H_2_O, an I:E ratio of 1∶1.5 and a FiO_2_ of 0.4. This ventilator is able to assess esophageal pressure (P_es_) by a balloon-tipped 8.0 Fr catheter which was placed into the esophagus posterior to the heart. Before application of muscle relaxants, a dynamic occlusion test was performed during spontaneous inspiratory efforts to assure that the esophageal catheter was in the correct position and changes of P_es_ reflected changes in airway pressure. Calculation of lung compliance (C_L_) was performed according to the following equation: C_L_ = (C_TRS_ • C_CW_)/(C_CW_ • C_TRS_); where C_TRS_ represents the compliance of the total respiratory system and C_CW_ represents the chest wall compliance calculated as the ratio of the exhaled tidal volume to the delta esophageal pressure (dP_es_) [17], [18]. Transpulmonary pressure (P_tp,es_), as the distending pressure of the lung, was measured during an end-inspiratory manoeuvre, reflecting the theoretical pressure difference between airway (P_aw_) and pleural pressure (P_pl_) using the following formula: (P_tp,es_ =  P_aw_−P_pl_). P_es_ was considered as a surrogate for pleural pressure (P_pl_) between the lung and the chest wall [19]. Normocapnia (pCO_2_ 35–40 mmHg) was achieved by adjusting the respiratory rate and end-tidal carbon dioxid was measured with an infrared absorption analyzer (suction rate 200 ml min^−1^; Sirecust 960, Siemens, Erlangen, Germany). For monitoring oxygen saturation, a pulse oximeter was placed on the ear (M-CaiOV, Datex-Ohmeda, Helsinki, Finland). Maintenance of anesthesia was performed by using propofol (4–6 mg kg^−1^ h^−1^) and sufentanil (0.3 µg kg^−1^ h^−1^). For the ensurance that changes in SVV and PPV reflected only the effects of positive pressure ventilation, muscle relaxation was provided by pancuronium (0.2 mg kg^−1^ h^−1^) to avoid spontaneous breathing efforts. Depth of anesthesia was monitored by Bispectral Index (BISXP, Aspect Medical Systems, Natick, MA, USA). We repeatedly performed pain stimuli like tail clamping to detect an inadequate depth of anesthesia and focused on the corneal reflex and lacrimation. If assessment suggested inadequate level of anesthesia, additional sufentanil and propofol was injected. During instrumentation, the pigs received an infusion of Ringer solution (6 ml kg^−1^ h^−1^). A heating blanket was used to avoid a drop in body temperature and to maintain temperature between 38.0 and 39.0°C. Cardiac rhythm was monitored by a standard lead II electrocardiogram.

### Hemodynamic Monitoring

For hemodynamic monitoring, a 7.5 Fr pulmonary artery catheter (Swan Ganz, CCO/VIP, 139HF75, Edwards Lifescience, Irvine, CA) was inserted percutaneously in the right internal jugular vein via an 8.5 Fr introducer for measurement of CVP, PAOP, pulmonary artery thermodilution cardiac output (CO_PAC_) and stroke volume (SV_PAC_) derived from pulmonary artery catheter. This catheter was advanced under continous pressure recording into wedge position and then connected to a CO monitor (Vigilance Monitor, Edwards Lifescience, Irvine, CA). A 5.0 Fr thermistor tipped catheter for thermodilution and pulse contour analysis was inserted percutaneously into the femoral artery (PV 2015L20, Pulsiocath, Pulsion Medical Systems AG, Munich, Germany) and was connected to the PiCCOplus Monitoring system (Version 6.0, Pulsion Medical Systems AG, Munich, Germany). This system allows discontinuous measurement of GEDV by transpulmonary thermodilution and continous measurement of SVV and PPV by pulse contour analysis. Thermodilution measurements were obtained by injecting 10 ml ice cold saline (≤8°C) through the central venous port of the pulmonary artery catheter to assess GEDV, CO_PAC_ and SV_PAC_ simultaneously. Regardless of the respiratory cycle, injections were performed at least three times. With respect to the preceding measurement, a difference of CO_PAC_ ≥15% was discarded and calibration repeated.

Ventilation induced percentage changes in pulse pressure and stroke volume were detected by the PiCCO monitoring system. SVV and PPV can be derived by the following equations:


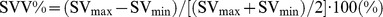
 [20]

and


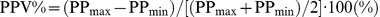
 [21].

Global End-diastolic Volume (GEDV) was calculated by transpulmonary thermodilution according to the following formula:


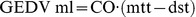
 [22]

where GEDV represents the sum of the right- and left-heart end-diastolic volumes. GEDV consists of the product of CO and the difference between mean transit time (mtt) and down-slope time (dst) measured by transpulmonary thermodilution. An 8.5 Fr introducer for volume infusion or blood withdrawal was placed into the femoral vein and a 22 Fr thoracic drainage tube (Portex, Smith Medical International Ltd., Kent, UK) was inserted at each side.

With the pig placed in the supine position pleural effusion was induced by intrapleural infusion of approximately 1500 ml warmed normal saline (50 ml kg^−1^) over 15 minutes into either side via the thoracic drainage tube and the magnitude of pleural effusions was repeatedly assessed by ultrasound (Vivid *i*, GE Healthcare, Munich, Germany). With respect to estimation of pleural effusion, the maximum end-expiratory distance between the parietal and visceral pleura was measured at the end of expiration and the estimated volume of the pleural effusion was then obtained by the following formula [23]: mm · 20 = ml of pleural effusion.

Magnitude of pleural effusions in each animal remained stable during the whole study period and was comparable in all animals.

### Experimental Protocol

**Figure 1:**
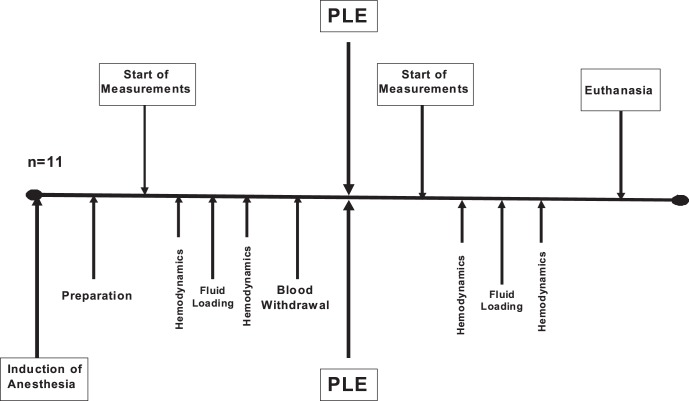
After induction of anesthesia and preparation, measurement of hemodynamics was performed before and after fluid loading. Following blood withdrawal and induction of pleural effusion, hemodynamics before and after fluid loading were determined again.

After establishing the monitoring and after each experimental step, at least 15 minutes were allowed for stabilization. Stable hemodynamic variables over a period of at least 5 minutes were a prerequisite before starting data collection. First, respiratory and hemodynamic variables were recorded after induction of anesthesia, defined as baseline. During data collection, three consecutive values for each hemodynamic and respiratory variable were noted down and calculation of the average was performed. Subsequently, a fluid bolus of 8 ml kg^−1^ 6% hydroxyethyl starch was administered over 10 minutes and measurement of fluid responsiveness was performed again. After data collection, baseline volume status was reestablished by stepwise withdrawal of 8 ml kg^−1^ blood through the venous femoral introducer. The blood was withdrawn into a sterile heparinized (5000 IE l^−1^) blood bag. Thereafter, normal saline was infused between the parietal and visceral pleura for induction of bilateral pleural effusion (approximately 1500 ml each side) and respiratory and hemodynamic variables were recorded. Subsequently, the withdrawn blood was given back to the animal, followed by data collection. Animals with an increase in stroke volume derived by pulmonary thermodilution of at least 15% after fluid challenge were considered to be fluid responsive (Responder) and those with less increase in stroke volume were considered to be Non-Responders. Before and after each fluid loading, SVV, PPV, GEDV, PAOP as well as SV_PAC_, CO_PAC_, arterial pressure and CVP were recorded simultaneously. Measurements were performed with the animal in supine position and in absence of heart rhythm disturbances. After completion of the trial period, animals were killed by an overdose of sufentanil, propofol and potassium chloride (Figure 1).

### Statistical Analysis

All data are given as mean±SD. Statistical comparisons were performed using commercially available statistics software (GraphPad Prism 5, GraphPad Software Inc., San Diego, CA, USA). A Kolmogorov-Smirnov test was used to test for Gaussian distribution. One way analysis of variance (ANOVA) was used for comparison of hemodynamic data at baseline and during pleural effusion, respectively. Receiver operator characteristic (ROC) curves were generated to investigate the ability of a variable to identify responders and non-responders. The optimal threshold value indicating maximum sensitivity and specificity was determined. Areas under the ROC curves were calculated and Pearson correlation for preload variables and subsequent changes in SV_PAC_ (ΔSV_PAC_) at baseline and during pleural effusion was performed. CVP, PAOP, SVV, PPV and GEDV at the different experimental stages were analyzed using one way analysis of variance. Paired t - test was used for comparison before and after fluid administration and unpaired t-test was used for comparison between responders and non-responders. P<0.05 was considered significant.

## Results

**Figure 2:**
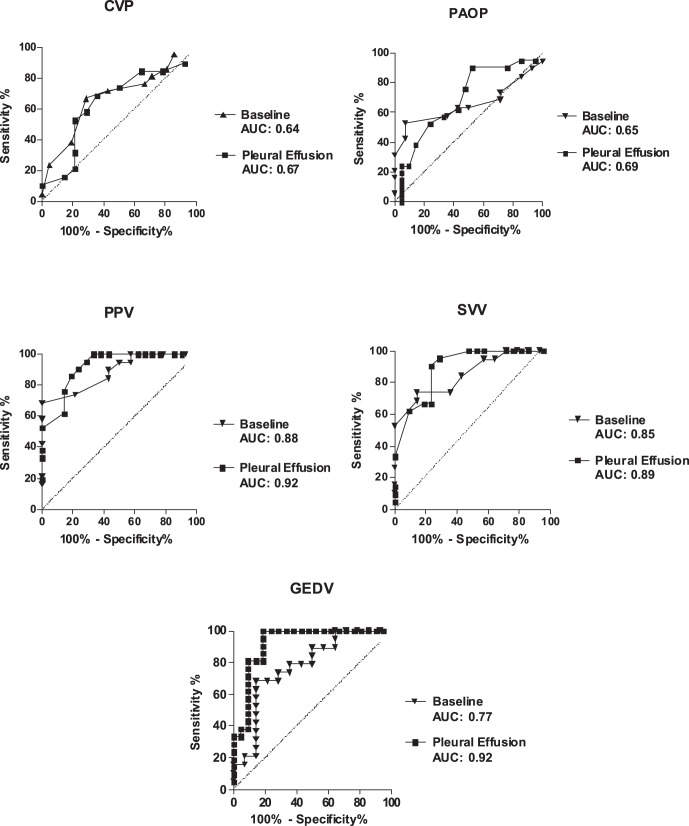
Prediction of fluid responsiveness at baseline and during pleural effusion: Area under the ROC curve (AUC). Ability of variables for predicting a ≥15% increase in stroke volume by pulmonary thermodilution (ΔSV_PAC_ ≥15%). PAOP, pulmonary artery occlusion pressure; CVP, central venous pressure; PPV, pulse pressure variation; SVV, stroke volume variation; GEDV, global end-diastolic volume. The straight line indicates line of identity. AUC = 0.5: prediction of fluid responsiveness not better than chance; AUC = 1.0: best prediction.

**Figure 3:**
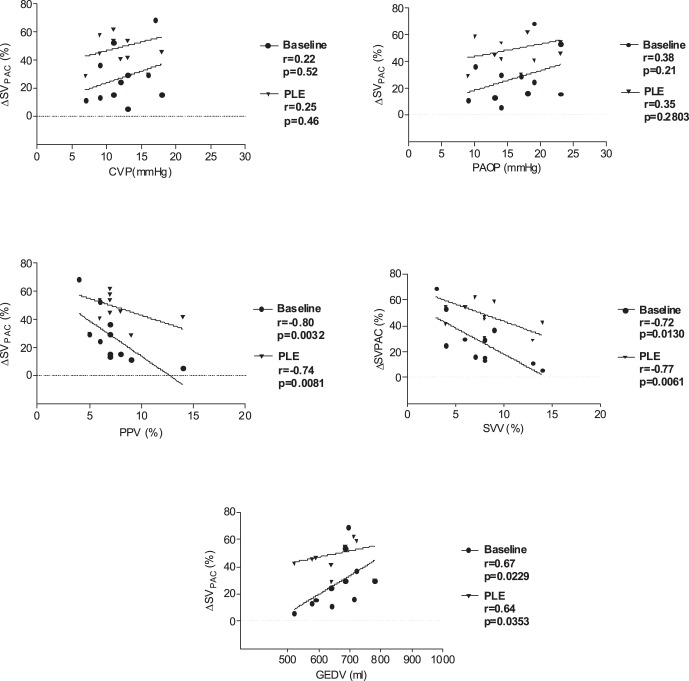
Correlation between dynamic and volumetric variables with percentage changes in stroke volume measured by pulmonary thermodilution (ΔSV_PAC_%) after fluid loading at baseline and during pleural effusion. CVP, central venous pressure; PAOP, pulmonary artery occlusion pressure; PPV, pulse pressure variation; SVV, stroke volume variation; GEDV, global end-diastolic volume.

Data of all 11 pigs were included into final analysis. Mean weight was 34±2 kg and we observed no hemodynamic instability requiring pharmacologic support during the measurements. Pleural effusion decreased lung compliance from 41±10 ml cmH_2_O^−1^ to 18±4 ml cmH_2_O^−1^ (p<0.05) and stroke volume by pulmonary thermodilution from 45.2±4.7 ml to 30.8±8.3 ml (p<0.05). There were 6 responders and 5 non-responders. Fluid loading increased stroke volume at baseline by 15±11% (p<0.05) and during pleural effusion by 23±17% (p<0.05). Hemodynamic and respiratory variables at baseline and during pleural effusion are presented in Table 1. At baseline, ROC analysis showed the best area under the curve (AUC) for PPV (AUC 0.88) with a 95% confidence interval of 0.77–0.99 and a p-value <0.001, followed by SVV (AUC 0.85; 0.72–0.98; p<0.001) and GEDV (AUC 0.77; 0.61–0.94; p<0.05). In contrast, CVP (AUC 0.64; 0.45–0.84; p = 0.17) and PAOP (AUC 0.65; 0.46–0.84; p = 0.14) were not able to predict an increase in SV_PAC_ ≥15%. During pleural effusion, AUC was 0.92 for PPV (0.84–1.00; p<0.001), 0.89 for SVV (0.79–0.99; p<0.001), 0.92 for GEDV (0.83–1.0; p<0.0001) and 0.69 for PAOP (0.53–0.85; p<0.05). Again, CVP (AUC 0.67; 0.51–0.84, p = 0.053) was not able to reliably predict fluid responsiveness (Figure 2). ROC analysis yielded threshold values for SVV and PPV to discriminate between responder and non-responder of 11.0% and 12.5% at baseline, and 14.5% and 15.5%, respectively, during pleural effusion (Table 2). Correlation between preload variables and percentage changes in stroke volume by pulmonary thermodilution (ΔSV_PAC_%) are shown in Figure 3.

**Table 1:** Hemodynamic and respiratory variables in Responder and Non-Responder before and after fluid loading during baseline and pleural effusion.

	Responder n = 6				Non-Responder n = 5			
Variable	BL - NV	PLE - NV	BL - FL	PLE - FL	BL - NV	PLE – NV	BL - FL	PLE - FL
**HR (min** ^−**1**^ **)**	123±15	138±23	1105	13421	11417	14423* ^,^ #	10917	13227
**MAP (mmHg)**	594	56±5	82±7^a^	82±3a,c	76±3	64±7	93±2* ^,^ Δ	87±4* ^,^ Δ
**SVR (dyne•s/cm^5^)**	892±225	1022±100	1155±245	1321±307^a^	960±174	1065±277	1173±173	1317±272*
**C_L_ (ml cmH_2_O** ^−**1**^ **)**	41±10	18±4a,b	39±9^c^	16±4a,b	38±13	16±4* ^,^ #	32±10Δ	15±3* ^,^ #
**C_CW_ (ml cmH_2_O** ^−**1**^ **)**	87±19	94±24	82±10	89±24	94±4	171±80* ^,^ #	897Δ	126±46#
**dP_es_ (cmH_2_O)**	8.0±1.4	12.9±5.7a,b	9.0±1.4^c^	13.3±1.4a,b	5.7±1.5	13.2±4.9* ^,^ #	7.0±2.9	13.5±3.1*
**P_tp,es_ (cmH_2_O)**	9.8±3.7	13.7±3.8a,b	8.3±3.9	14.3±4.6a,b	10.7±4.0	14.2±4.3* ^,^ #	8.5±4.7	15.8±3.1* ^,^ #
**P_AW peak_ (cmH_2_O)**	24±3	36±3a,b	24±3^c^	39±4a,b	25±4	40±5* ^,^ #	26±3Δ	41±4* ^,^ #
**P_AW mean_ (cmH_2_O)**	12±2	15±1	12±2	15±1	12±1	16±1	12±1	16±1
**V_T_ (ml)**	350±56	344±57	343±49	334±42	347±42	341±46	340±48	337±47
**PEEP (cmH_2_O)**	5.1±2.4	5.9±0.5	5.2±2.3	5.1±1.4	5.8±0.9	5.2±0.4	5.5±1.3	5.1±0.5
**CO_PAC_ (l min** ^−**1**^ **)**	5.1±0.7	3.9±0.7a,b	6.2±0.4a,c	5.3±1.1b,c	6.2±1.2	4.0±0.9* ^,^ #	6.5±1.8Δ	4.1±1.3* ^,^ #
**SV_PAC_ (ml min** ^−**1**^ **)**	45.2±4.7	30.8±8.3a,b	52.3±5.1^a^	41.7±9.1b,c	52.3±8.2	31.5±8.9* ^,^ #	53.8±7.4Δ	31.2±9.7* ^,^ #
**CVP (mmHg)**	7.2±2.8	12.0±3.4^a^	9.2±3.2	13.3±2.6a,b	7.5±5.7	10.8±2.6	10.2±5.8	12.8±2.7*
**PAOP (mmHg)**	10.3±2.3	13.3±1.6^a^	12.5±2.3	15.7±2.9^a^	10.0±5.3	15.0±4.6*	13.0±4.5	16.2±1.9* ^,^ #
**PPV (%)**	15.6±3.7	20.6±8.2^b^	11.0±1.7a,c	10.7±5.3a,c	8.6±3.1	9.4±7.1#	10.0±2.6Δ	14.0±7.7* ^,^ #
**SVV (%)**	13.7±5.3	17.5±6.9	10.7±3.2 c	10.4±3.1^c^	8.2±2.9	9.5±4.7#	9.4±3.9	12.5±3.7* ^,^ #
**GEDV (ml)**	635±61	496±87^b^	694±43a,c	591±83^b^	743±93	641±87* ^,^ #	764±82* ^,^ Δ	715±103Δ

BL - NV, baseline normovolemia; PLE - NV, pleural effusion normovolemia; BL - FL, baseline fluid loading; PLE - FL, pleural effusion fluid loading; HR, heart rate; MAP, mean arterial pressure; SVR, systemic vascular resistance; C_L_, lung compliance; C_CW_, chest wall compliance; dP_es_, delta esophageal pressure; P_tp,es_, transpulmonary pressure measured with an esophageal balloon; P_AW peak_, end-inspiratory airway pressure; P_AW mean_, mean airway pressure; V_T_, tidal volume; PEEP, positive end-expiratory pressure; CO_PAC_, cardiac output derived from pulmonary thermodilution; SV_PAC_, stroke volume derived from pulmonary thermodilution; CVP, central venous pressure; PAOP, pulmonary artery occlusion pressure; PPV, pulse pressure variation; SVV, stroke volume variation; GEDV, global end-diastolic volume; Values are given as mean ±SD; Responder:

a p<0.05 (vs. BL - NV);

b p<0.05 (vs. BL - FL);

c p<0.05 (vs. PLE – NV); Non-Responder:

* p<0.05 (vs. BL - NV);

# p<0.05 (vs. BL - FL);

Δ p<0.05 (vs. PLE – NV).

**Table 2:** Area under the Receiver Operating Characteristic Curve showing the ability of preload variables to predict an increase in stroke volume generated by pulmonary thermodilution ≥15% at baseline and during pleural effusion.

	BL					PLE				
	CVP(mmHg)	PAOP(mmHg)	GEDV(ml)	PPV (%)	SVV (%)	CVP(mmHg)	PAOP(mmHg)	GEDV (ml)	PPV (%)	SVV (%)
**AUC**	0.64	0.65	0.77	0.88	0.85	0.67	0.69	0.92	0.92	0.89
**95% CI**	0.45–0.84	0.46–0.84	0.61–0.94	0.77–0.99	0.72–0.98	0.51–0.84	0.53–0.85	0.83–1.00	0.84–1.00	0.79–0.99
**Threshold value**	n.a.	n.a.	>703	<12.5	<11.0	n.a.	>14.5	>584	<15.5	<14.5
**Sensitivity (%)**	68	63	79	74	74	71	76	95	95	95
**Specificity (%)**	64	57	64	79	86	57	52	81	71	71
**P-value**	0.17	0.14	0.007	0.0002	0.0006	0.053	0.034	<0.0001	<0.0001	<0.0001

BL, baseline; PLE, pleural effusion; AUC, area under the curve; 95% CI, 95% confidence interval; CVP, central venous pressure; PAOP, pulmonary artery occlusion pressure; GEDV, global end-diastolic volume; PPV, pulse pressure variation; SVV, stroke volume variation; n.a., not assessed.

## Discussion

Main findings of our experimental animal investigation are as follows:

In presence of bilateral pleural effusion and during different loading conditions, the dynamic and volumetric variables SVV, PPV and GEDV were able to predict a percentage change in stroke volume. The changed threshold values, however, indicate that for proper interpretation of these variables, information regarding the presence of pleural effusion is important.

Appropriate perioperative fluid loading according to the patient’s individual needs has been shown to reduce mortality and length of stay on the intensive care unit [1]. Beside estimation of beat-to-beat stroke volume and cardiac output by pulse contour analysis, ventilation induced dynamic variables of fluid responsiveness generated by less invasive monitoring systems have also gained increasing interest for guiding fluid therapy. However, recent studies could demonstrate that fluid loading is still commonly based on pressure derived variables such as CVP and PAOP [24] which have been shown to be not suitable for prediction of fluid responsiveness [3].

Accordingly, also in our study CVP failed to reliably predict an increase of stroke volume during fluid loading at baseline and in presence of pleural effusion. Interestingly, in presence of pleural effusion, PAOP as a static cardiac filling pressure achieved statistical significance in predicting fluid responsiveness. Although several studies have repeatedly demonstrated poor reliability of filling pressures to predict fluid responsiveness [25], a possible explanation of our findings may be found considering changes in pleural pressure as the principal determinant for maintenance of right- and left-heart blood flow [26]. Given the curvelinear left ventricular pressure-volume relationship, at low cardiac preload the increase in volume is higher than the increase in pressure after fluid loading, while the opposite is true at higher cardiac filling, i.e. with decreased ventricular compliance, pressure derived variables may gain predictive power. With a large pleural effusion the compliance of the left ventricle may decrease and may thus have enabled PAOP to become predictive. This explanation is supported by a recent study demonstrating PAOP being superior to GEDVI in patients with left ventricular dysfunction [27].

Changes in intrathoracic pressure and consecutive changes in venous return and left ventricular preload were identified as the physiological background underlying both SVV and PPV [28]. However, recent studies demonstrated that these physiological principles may be diminished or even abolished during open chest conditions, spontaneous breathing efforts, low tidal volume ventilation or high respiratory rate [7], [8], [29]. In this context, changes in pleural pressure caused by pleural effusions may also interact with the reliability of SVV and PPV. However, currently there are no data available concerning the reliability of dynamic variables in the presence of pleural effusion.

Recently, an animal study investigated the influence of an unilateral pleural effusion on respiratory mechanics and chest wall compliance [30]. Interestingly, the authors obtained a progessive reduction in lung compliance by pleural effusion which was associated with enlargement of chest wall expansion, respectively increased chest wall compliance. These findings are in agreement with our results, as we also observed decreased lung compliance and consecutive increased chest wall compliance in presence of a positive end-expiratory pressure (5 cmH_2_O) and pleural effusion especially in non-responder. With respect to the hemodynamic effect of pleural effusion, recent animal investigations could demonstrate that induction of a pleural effusion up to 40 ml kg^−1^ did not affect hemodynamic variables such as cardiac output and arterial pressure but increased PAOP and CVP. In contrast, pleural effusion up to 80 ml kg^−1^ was associated with severe reduction in cardiac output and arterial pressure, thereby causing half of the animals to die [31]. Accordingly, we observed a significant reduction in cardiac output and stroke volume by pulmonary thermodilution during a pleural effusion of 50 ml kg^−1^, as well as a significant increase in static cardiac filling pressures, but none of the animals died after initiation of pleural effusion (Table 1). Interestingly, chest wall compliance did not change significantly in the responder group, whereas the non-responders revealed significant changes in chest wall compliance. An explanation for these findings could be a pronounced buffering effect by chest wall expansion, dissociating pleural and intrathoracic pressure in non-responders [30], [32]. In contrast, less chest wall expansion possibly results in a more compressive effect on mediastinal organs by pleural fluid volume and therefore could enhance the responsiveness to fluid loading. This hypothesis is supported by lower GEDV values in the responder group, indicating less ventricular filling due to an increasing compressive effect by pleural effusion. With respect to volume responsiveness and reduced ventricular filling, a recent study obtained a significant increase in cardiac output and stroke volume by fluid challenges in patients with reduced ventricular volume caused by cardiac tamponade [33].

Several investigations demonstrated transpulmonary pressure as a useful tool to estimate and improve respiratory mechanics in critically ill patients [34]. With respect to transpulmonary pressure in our study, determined as the difference between airway and pleural pressure, we could not obtain a significant difference between responders and non-responders.

Interestingly, in presence of pleural effusion, ROC analysis yielded reliable prediction of fluid responsiveness by SVV and PPV compared with baseline measurements in responders. An explanation for these results might be the underlying ventilation induced variation in intrathoracic pressure, which accounts for SVV and PPV and may be increased by pleural fluids. In this context, a recent animal study, investigating hemodynamic changes in presence of pericardial and pleural effusions, obtained better toleration of elevated intrapericardial pressure in presence of pleural effusion [35]. This could be explained by an enhancement of ventilatory swings in intrathoracic pressure associated with an increase of blood flow toward the heart cavities. In the present study we observed a significant increase of threshold values for SVV and PPV which play an important role for the clinician in the decision making process if the patient needs fluid or not. These shifted threshold values for PPV might even be advantageous, due to the fact that recent investigations could demonstrate poor prediction of fluid responsiveness by PPV values between 9–13%, exhibiting the same number of responders and non-responders [36].

With respect to the reliability of the static volumetric variable GEDV to predict fluid responsiveness, recent literature remains controversial [4]. This is due to the fact that static volumetric variables on principle do not reflect ventricular compliance and contractility, respectively, and therefore merely reflect preload but not preload responsiveness. However, we obtained reliable prediction of fluid responsiveness by GEDV at baseline as well as in presence of pleural effusion during different loading conditions. Our results are in line with other investigations dealing with volumetric variables and prediction of stroke volume increase [5], [37], [38]. These authors obtained sufficient accuracy of GEDV to predict a percentage change in stroke volume, even during elevated intraperitoneal pressure and in patients with septic shock. Increased intraperitoneal pressure was associated with reliable prediction of fluid responsiveness by PPV and GEDV, in contrast to SVV, and furthermore, threshold values for PPV were significantly increased. A recent meta-analysis, however, suggested GEDVI to be a poor predictor of fluid responsiveness [4]. A possible explanation for the differing results could be the underlying relationship between preload and stroke volume. As described by the Frank-Starling mechanism the greater the preload the greater should be the increase in stroke volume. However, as the slope of the Frank-Starling curve depends on contractility, increasing preload will not automatically lead to an increase in stroke volume [39]. In a recent clinical investigation, ability of GEDV to predict fluid responsiveness depended on left ventricular function with left ventricular dysfunction abolishing prediction of fluid responsiveness [27].

Several limitations of our study should be noted. First, we investigated healthy pigs with normal cardiac and pulmonary function certainly not exhibiting lung injuries like pneumonia compared to critically ill patients. Therefore our results cannot directly transferred to critically ill patients, suffering from diseases causing pleural effusion. Chronic diseases associated with pleural effusions may interact by stiffening the lung and the chest wall with consecutive reduction of chest wall compliance. Therefore, influence of pleural effusion on variables of fluid responsiveness should be investigated in humans.

In conclusion, the present experimental animal study demonstrated reliable indication of fluid responsiveness by GEDV, SVV and PPV even in presence of large pleural effusions. However, ROC-analysis yielded increased threshold values for SVV and PPV to discriminate between responder and non-responder in presence of pleural effusion. As only healthy pigs with normal cardiac and pulmonary function were investigated, the present results cannot be generalized and extrapolated to critical ill patients.
